# Diffusion Tensor MR Imaging Evaluation of Callosal Abnormalities in Schizophrenia: A Meta-Analysis

**DOI:** 10.1371/journal.pone.0161406

**Published:** 2016-08-18

**Authors:** Chuanjun Zhuo, Mei Liu, Lina Wang, Hongjun Tian, Jinsong Tang

**Affiliations:** 1 Department of Psychiatry Functional Neuroimaging Laboratory, Tianjin Mental Health Centre, Tianjin Anding Hospital, Tianjin, China; 2 Institute of Mental Health, the Second Xiangya Hospital of Central South University, Changsha, Hunan, China; 3 Key Laboratory of Psychiatry and Mental Health of Hunan Province, The National Clinical Research Center for Psychiatric and Psychological Diseases, Changsha, Hunan, China; Maastricht University, NETHERLANDS

## Abstract

Widespread white matter (WM) abnormalities have been found in patients with schizophrenia. Corpus callosum (CC) is the key area that connects the left and right brain hemispheres. However, the results of studies considering different subregions of the CC as regions of interest in patients with schizophrenia have been inconsistent. To obtain a more consistent evaluation of the diffusion characteristics change of the corpus callosum (CC) related to schizophrenia. A meta-analysis involving fractional anisotropy (FA) values in the CC of 729 schizophrenic subjects and 682 healthy controls from 22 studies was conducted. Overall FA values in the CC of the schizophrenic group were less than that of the healthy control group [weighted mean difference (WMD) = -0.021,P< 0.001]. So were the FA values in the genus region (WMD = -0.019, P< 0.001) and the splenium region (WMD = -0.020, P< 0.001) of the CC respectively. The FA reduction was also significant in subjects with chronic schizophrenia (WMD = -0.032, P< 0.001) and first-episode schizophrenia (WMD = -0.014, P = 0.001). In present study, we demonstrated an overall FA decrease in the CC of schizophrenic patients. In the two subgroup analyses of the genu vs splenium region and chronic vs first-episode schizophrenia, the decrease of all groups was significant. Further studies with more homogenous populations and standardized DTI protocols are needed to confirm and extend these findings.

## Introduction

More than two hundred million people around the world are suffering from schizophrenia. Schizophrenia is an incapacitating psychiatric disorder characterized by disturbances in perception, cognition, and emotion, as well as an altered sense of self and abnormal behavior patterns. People with schizophrenia experience delusions and auditory hallucinations [1]. The risk of early mortality among schizophrenic patients is more than double that of the general population. It is attributed to a variety of conditions, including sequelae of pathogen infections, cardiovascular diseases and metabolic disorders (http://www.who.int/).

Physiologically, schizophrenia is associated with an impaired white matter (WM) connectivity[1]. It has been suggested that some of the symptoms of schizophrenia may be due to observed abnormalities of the corpus callosum (CC)[2], a massive bundle of WM tracts that mediates the cross-talk between the cerebral hemispheres. Indeed, patients with schizophrenia have been found to have a smaller CC than healthy subjects[3].

Diffusion tensor imaging (DTI) provides a convenient way of studying brains of schizophrenic patients and healthy controls. DTI is a non-invasive magnetic resonance (MR) method of assessing water diffusion tendencies in WM structures[4]. The diffusivity isrelated to boundaries created by neural tracts and myelin sheaths and thus can be used as a marker for WM tract integrity[5].Many properties can be calculated from the data obtained by DTI, such as mean diffusivity (MD), apparent diffusion coefficient(ADC),Fractional Anisotropy (FA) and the axial/radial diffusivity[6].

The findings of a recent meta-analysis of DTI studies suggested that schizophrenia may involve an abnormality of the splenium region of the CC in particular and thus perhaps altered posterior interhemispheric connectivity[7].Widespread WM abnormalities have been found in patients with first-episode or chronic schizophrenia[1, 8–12].

In this study, we conducted a meta-analysis of WM diffusion alterations in the CC of schizophrenic patients, both chronic and first-episode cohorts, compared with observations in healthy control subjects. Fractional anisotropy (FA) values of collated studies were submitted to statistical analysis.

## Materials and Methods

The protocol for this meta-analysis supporting checklist and flow diagram are available as supporting information (S1 File, S1 Checklist)

### Data sources and searches

We performed a comprehensive search on electronic database including PubMed, Embase and Cochrane for relevant studies published from the earliest indexing year to June 15^th^, 2016. Document type was restricted to article and publication language was restricted to English.

In order not to miss out papers that did not mention the CC in the title or abstract but covered analysis as part of the white matter or the whole brain in the full text, we extended our search to include white matter and brain. Eventually, the key words used to define relevant articles included: diffusion tensor imaging, schizophrenia, corpus callosum, white matter and brain. Besides the subject terms, some other related free terms were also used. See **S2 File** for detailed search strategy.

### Study inclusion criteria

Studies were evaluated for inclusion and the criteria were the followings: (1) subjects with schizophrenia were compared to unrelated healthy controls; (2) the diagnostic criteria for schizophrenia were clearly stated; (3) they obtained DTI data with a 1.5-T or 3.0-T scanner (sufficient power to meet advanced technical standards for clinical brain imaging); (4) at least a single FA value in the whole CC and/or the genu or splenium of the CC was reported.

### Data extraction

From all eligible studies, we extracted four types of data: study characteristics (study name, author, publication year, numbers of patients and controls), technical parameters of DTI (field strength, b values, directions, slices, slice thickness, data analysis methods), moderator variables (schizophrenia type, age, gender, handedness, medication use, duration of illness) and dependent variables (FA means, FA standard deviations).

### Statistical analysis

Meta-analyses of continuous outcomes (FA values) were performed with weighted mean differences (WMDs)in Stata software (Version 14.0 Stata Inc., College Station, TX). The DerSimonian and Laird random-effects model was employed with a 95% confidence interval (CI) to account for measurement variability among the included studies. We pooled and compared FA data from schizophrenic subjects versus from healthy controls. Additionally, we pooled and statistically compared FA data of genu region with splenium region and data of first-episode schizophrenia subjects with chronic schizophrenia subjects. The random-effects model was used, and inconsistency was evaluated by the I-squared index. The resultant data are presented in forest plots. Publication bias was assessed by Egger’s method. For all comparisons, *P* values < 0.05 was considered the criterion of statistical significance.

## Results

### Included studies and characteristics

**Fig 1:**
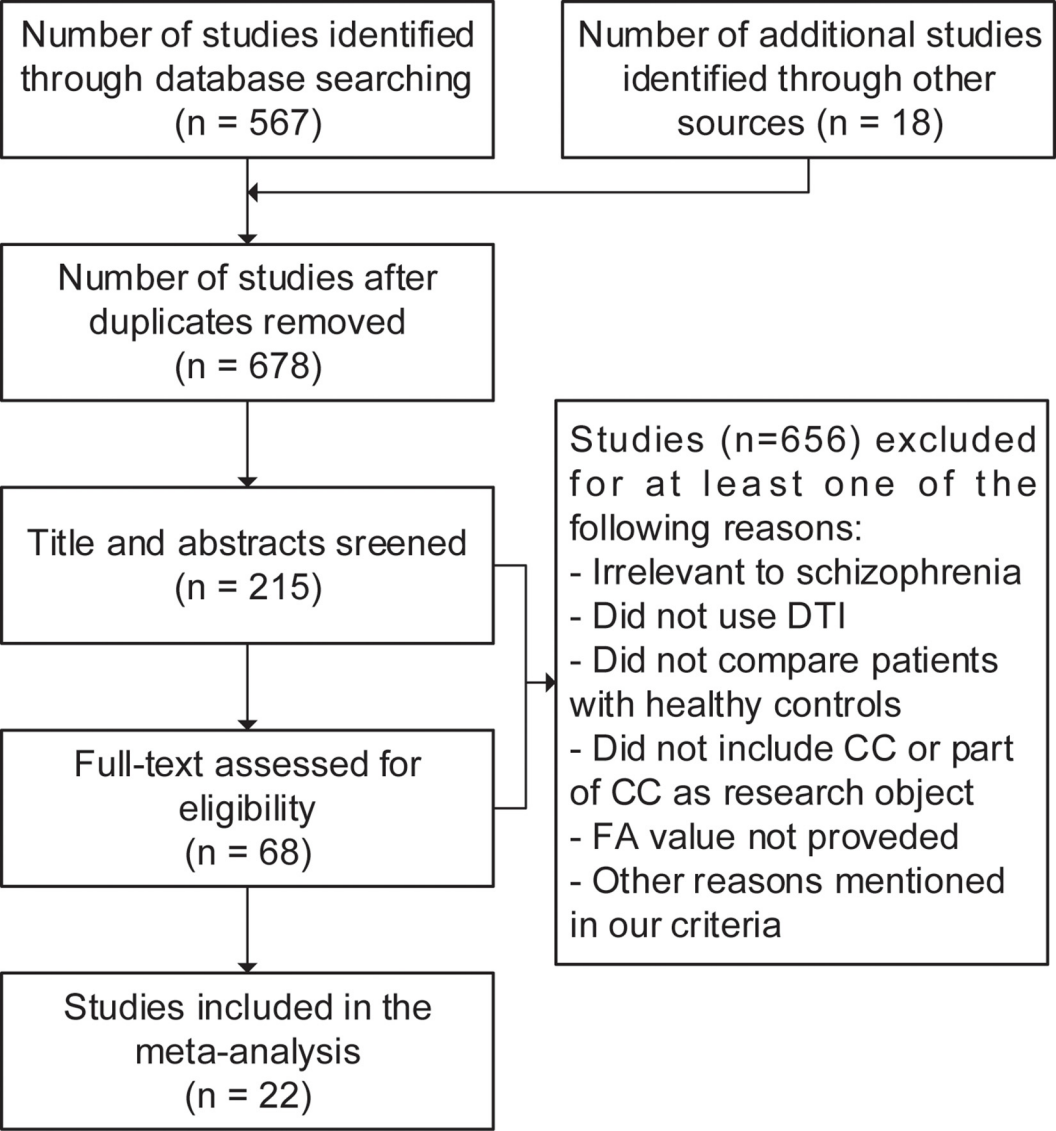
Screening and selection process for articles.

Our search of the three databases identified 678 citations after excluding duplicates with computer assistance and manually. We scanned the title and abstract or the full text of articles and excluded 610 papers obviously unrelated to our purpose. We examined the remaining 68 articles carefully and 22[13–34]meeting our criteria were included in this meta-analysis finally (**Fig 1**).

The study group characteristics are summarized in **Table 1**. The meta-analysis included a total of 729 schizophrenia subjects [FA values from 1229 volumes of interest (VOIs)] and 682 healthy controls (1314 VOIs) from 22 studies (42 datasets). In general, the number of male subjects is larger than that of female subjects. Most of the subjects are right handed. The medication use of Schizophrenic patients is different among the studies. Most of the studies include patients with medications and some focus on drug-naive patients.

**Table 1:** Study characteristics of included articles.

Study	Group	Type	Number	Mean Age (year)	Gender (male%)	Handedness (right%)	Medication Use	Duration of Illness (month)
Foong 2000	Schizophrenics	NS	20	37.7	75.0%	95.0%	100%	165
	Controls	-	25	33.8	64.0%	96.0%	-	-
Sun 2003	Schizophrenics	NS	30	27.4	60.0%	100.0%	100%	279.6
	Controls	-	19	25.7	63.2%	100.0%	-	-
Kubicki 2005	Schizophrenics	C	21	18–55^a^	NS	100.0%	NS	NS
	Controls	-	26	18–55^a^	NS	100.0%	-	-
Price 2005	Schizophrenics	F	20	25.0	70.0%	85.0%	80%	NS
	Controls	-	29	28.1	37.9%	93.1%	-	-
Cheung 2008	Schizophrenics	F	25	28.5	44.0%	87.0%	0%	6
	Controls	-	26	28.2	50.0%	95.0%	-	-
Friedman 2008	Schizophrenics	C	40	45.2	70.0%	90.0%	97.50%	272.1
	Controls	-	40	45.2	70.0%	92.5%	-	-
	Schizophrenics	F	40	25.8	75.0%	95.0%	97.50%	11.6
	Controls	-	39	25.4	72.0%	82.1%	-	-
Peters 2008	Schizophrenics	F	10	21.2	100.0%	80.0%	100%	10.8
	Controls	-	10	21.1	100.0%	80.0%	-	-
Rotarska-Jagiela 2008	Schizophrenics	C	24	39.0	50.0%	100.0%	95.90%	151
	Controls	-	24	39.2	50.0%	100.0%	-	-
Gasparotti 2009	Schizophrenics	F	21	28.5	52.4%	100.0%	0%	19.71
	Controls	-	21	27.4	61.9%	100.0%	-	-
Kanaan 2009	Schizophrenics	NS	76	30.9	86.9%	100.0%	85.50%	48 (median)^b^
	Controls	-	76	30.5	85.5%	100.0%	-	-
Mandl 2010	Schizophrenics	C	40	26.8	75.0%	92.5%	100%	25.1
	Controls	-	40	28.0	72.5%	87.5%	-	-
Kitis 2011	Schizophrenics	NS	25	38.1	56.0%	NS	100%	125.8
	Controls	-	17	33.4	52.9%	NS	-	-
Kong 2011	Schizophrenics	F	15	24.3	66.7%	100.0%	Most^c^	8.3
	Schizophrenics	C	15	24.3	66.7%	100.0%	Most^c^	45.4
	Controls	-	15	24.2	66.7%	100.0%	-	-
Henze 2012	Schizophrenics	F	13	17.1	61.5%	84.7%	100%	7.55
	Controls	-	13	17.6	61.5%	100.0%	-	-
Knöchel 2012	Schizophrenics	C	16	37.6	56.3%	100.0%		164.5
	Controls	-	16	39.3	NS	100.0%	-	-
Kunimatsu 2012	Schizophrenics	NS	39	29.5	48.7%	100.0%	100%	84.2
	Controls	-	40	30.0	50.0%	100.0%	-	-
Lee 2013	Schizophrenics	F	17	21.5	76.5%	100.0%	70.60%	10.9
	Controls	-	17	23.1	70.6%	100.0%	-	-
Asami 2014	Schizophrenics	C	24	44.4	100.0%	71.0%	87.50%	246.0
	Controls	-	25	40.4	100.0%	78.0%	-	-
Collinson 2014	Schizophrenics	F	65	29.3	70.8%	Most	100%	27.7
	Schizophrenics	C	48	37.0	70.8%	Most	100%	144.5
	Controls	-	73	32.4	64.4%	90.4%	-	-
Kochunov 2014	Schizophrenics	NS	30	40.1	70.0%	NS	76.70%	231.6
	Controls	-	40	41.9	57.5%	NS	-	-
Harms 2015	Schizophrenics	NS	25	24.2	80.0%	NS	100%	62.4
	Controls	-	18	21.4	55.6%	NS	-	-
Nugent 2015	Schizophrenics	NS	30	39.4	73.0%	NS	90%	NS
	Controls	-	33	38.9	52.0%	NS	-	-

NS, not specified; -, not applicable; C, chronic; F, first-episode.

^a^ The article does not refer to the mean of age but the range.

^b^ The article only refers to the median of the duration instead of the mean.

^c^ The medication use of chronic and first-episode patients is described as a whole and cannot be separated.

The imaging parameters of the included studies are summarized in **Table 2**. Briefly, a 3-T MRI scanner was used in 12 of the included studies and a 1.5-T MRI scanner was used in 10 of the studies. A maximum b factor of 1000 s/mm^2^was most common. The DTI directions varied considerably, ranging from 6to 64. Scan thicknesses were mostly in the range of 2.5–5 mm.

**Table 2:** Summary of imaging parameters.

Study	Method	Field strength (T)	B value (s/mm^2^)	Scan time (minutes)	Directions	Slices	Slice thickness (mm)
Foong 2000	ROI	1.5	0–700	NS	7	NS	5
Sun 2003	ROI	1.5	0–1000	NS	25	NS	5
Kubicki 2005	Voxel-based	1.5	0–1000	NS	6	31–35	4
Price 2005	ROI	1.5	0–700	NS	7	NS	5
Cheung 2008	ROI	1.5	0–1200	NS	25	NS	5
Friedman 2008	ROI	3.0	0–10000	50	12	208	0.82
Peters 2008	Tractography	3.0	0–1000	6	16	NS	2.2
Rotarska-Jagiela 2008	ROI	3.0	0–1000	25	NS	40	2
Gasparotti 2009	Tractography	1.5	0–1000	NS	6	29	5
Kanaan 2009	Voxel-based	1.5	0–1300	NS	64	60	2.5
Mandl 2010	ROI	1.5	0–1000	7	32	160–180^a^	1.2
Kitis 2011	Tractography	1.5	0–700	NS	60	NS	2.2
Kong 2011	Voxel-based	1.5	0–1000	NS	13	30	4
Henze 2012	ROI	1.5	0–1000	NS	6	50	2.5
Knöchel 2012	Voxel-based	3.0	0–1000	NS	6	176	1
Kunimatsu 2012	Tractography	1.5	0–1000	5.6	6	30	5
Lee 2013	TBSS	3.0	0–900	NS	51	85	1.7
Asami 2014	TBSS	3.0	0–900	NS	51	85	1.7
Collinson 2014	Voxel-based	3.0	0–800	NS	15	180	0.9
Kochunov 2014	TBSS	3.0	0–700	9	64	50	3
Harms 2015	ROI	3.0	0–800	5	30	NS	2
Nugent 2015	TBSS	3.0	0–700	9	64	50	3

ROI, region of interest; TBSS, Tract-Based Spatial Statistics; NS, not specified.

^a^The article only provides a range instead of fixed value.

### Overall

**Fig 2:**
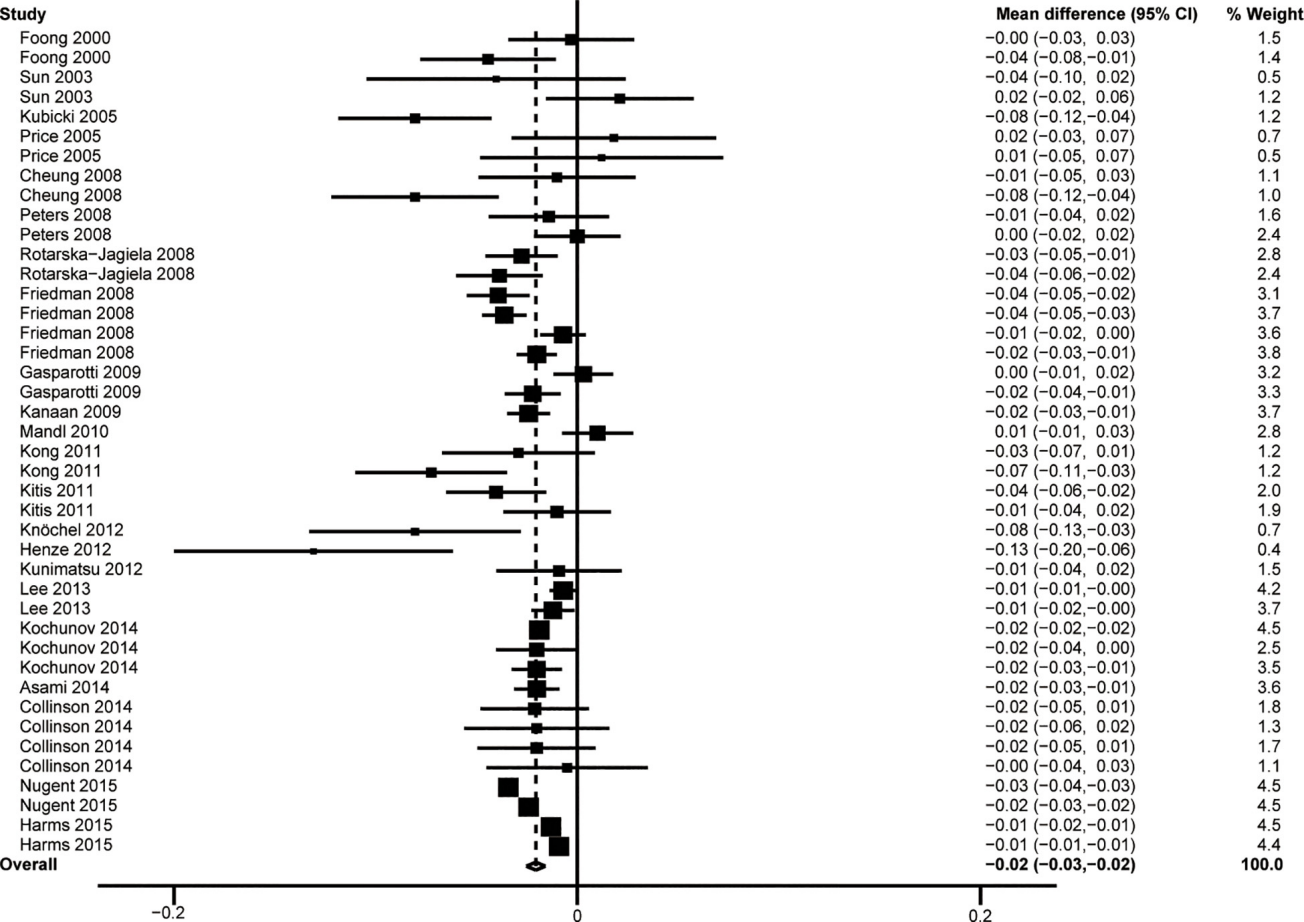
Results of the overall analysis.

**Fig 3:**
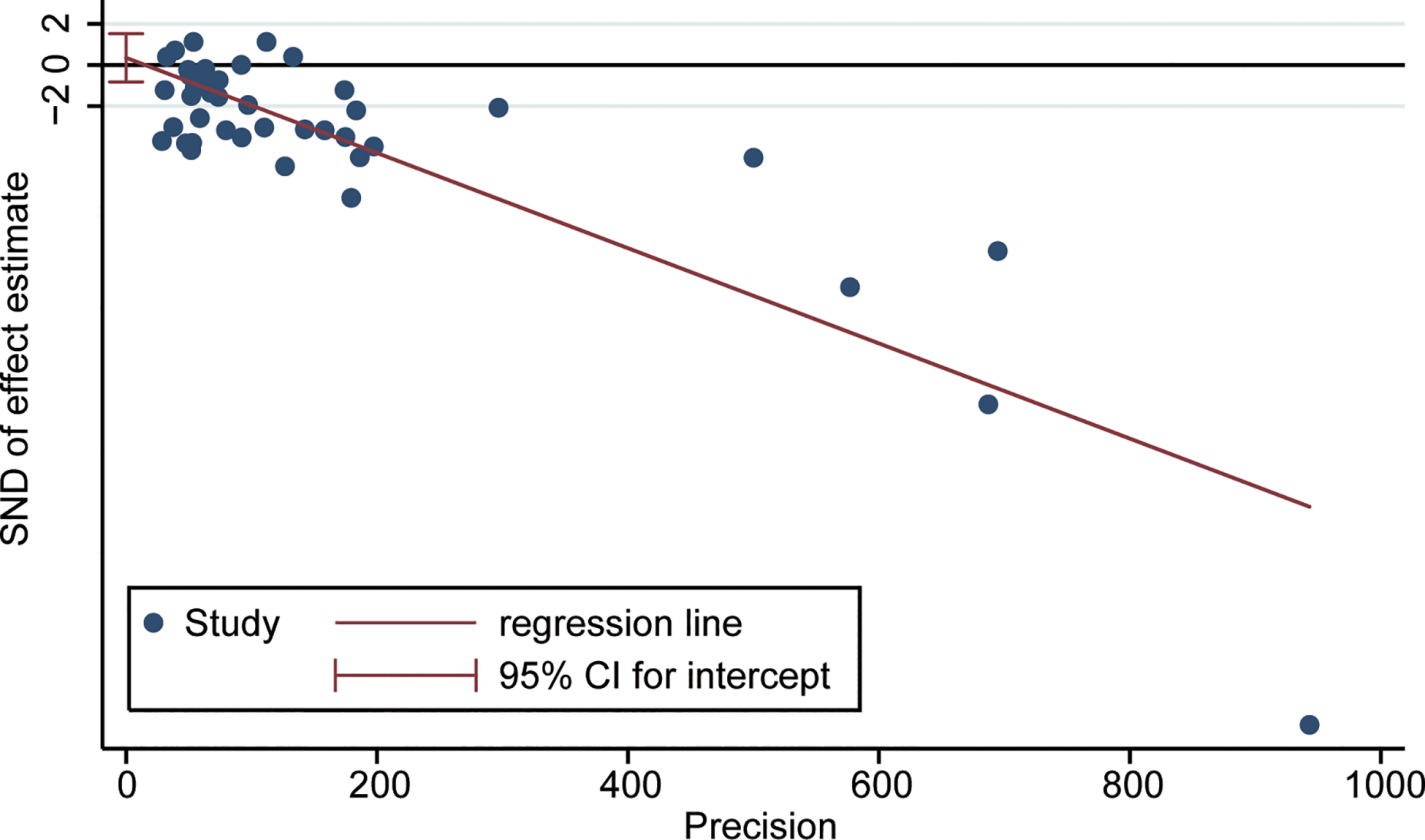
Results of publication bias analysis. Bias was analyzed with Egger’s method. t = 0.6, *P* = 0.55.

See S3 File for detailed FA data extracted from all included studies. The overall analysis revealed lower FA values for WM in the CC of subjects with schizophrenia than of unrelated healthy controls (WMD = -0.020, 95%CI = -0.025–-0.016, Z = 8.55, *P*< 0.001,*I*^2^ = 88.0%) and the details was presented in **Fig 2**. The result of Egger’s test (**Fig 3**) indicated that there was no publication bias (t = 0.6, *P* = 0.55).

### Genu vs splenium

**Fig 4:**
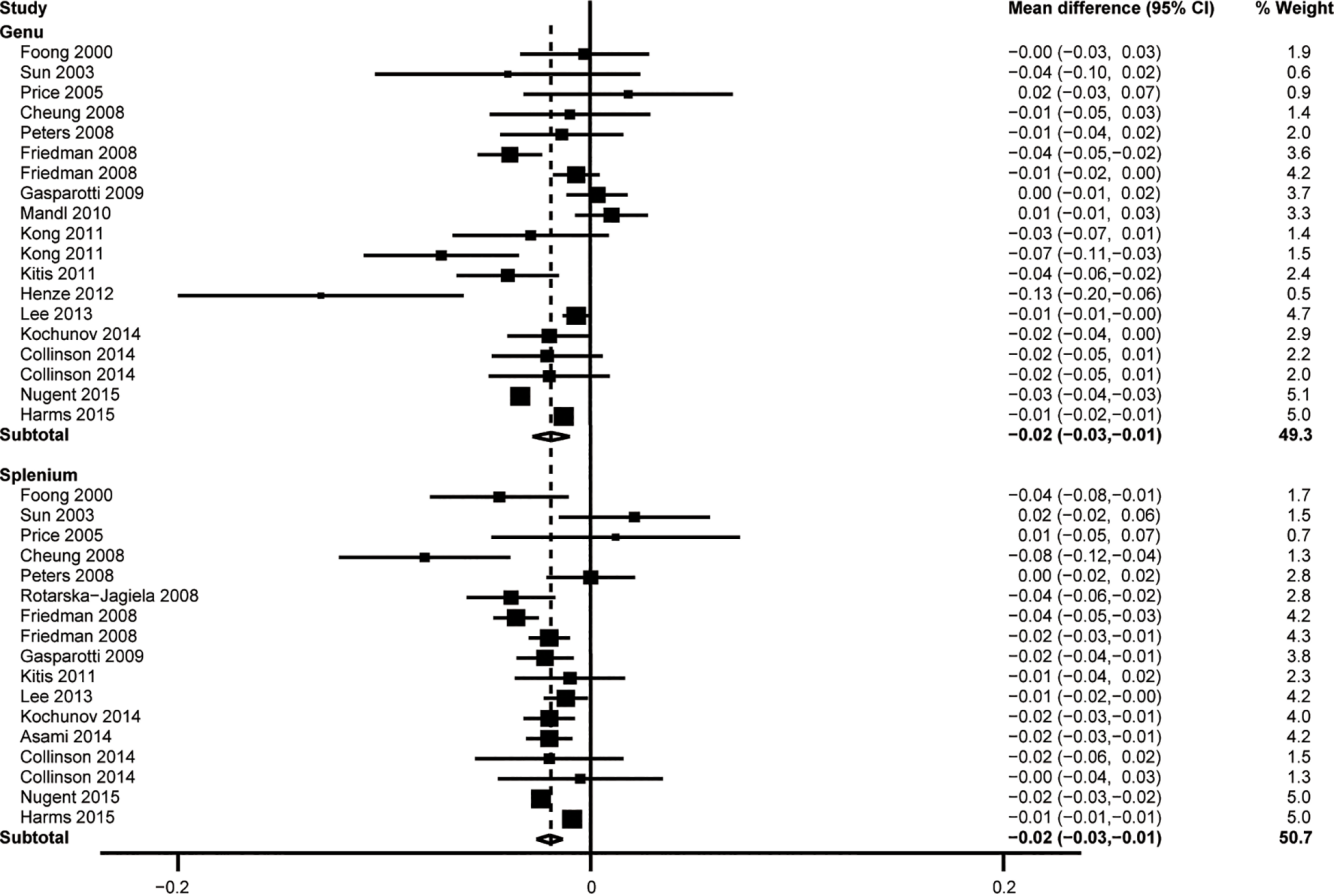
Results of subgroup analysis of different parts of the CC.

Likewise, subgroup analyses revealed lower FA values in boththe genu (19 comparisons; WMD = -0.019, 95%CI = -0.028–0.010, Z = 4.19, *P* < 0.001, *I*^2^ = 92.3%) and splenium (17 comparisons; WMD = -0.020, 95%CI = -0.026–0.014, Z = 6.16, *P*< 0.001, *I*^2^ = 77.6%) regions of the CC of schizophrenic subjects (**Fig 4**).

### First-episode vs chronic

**Fig 5:**
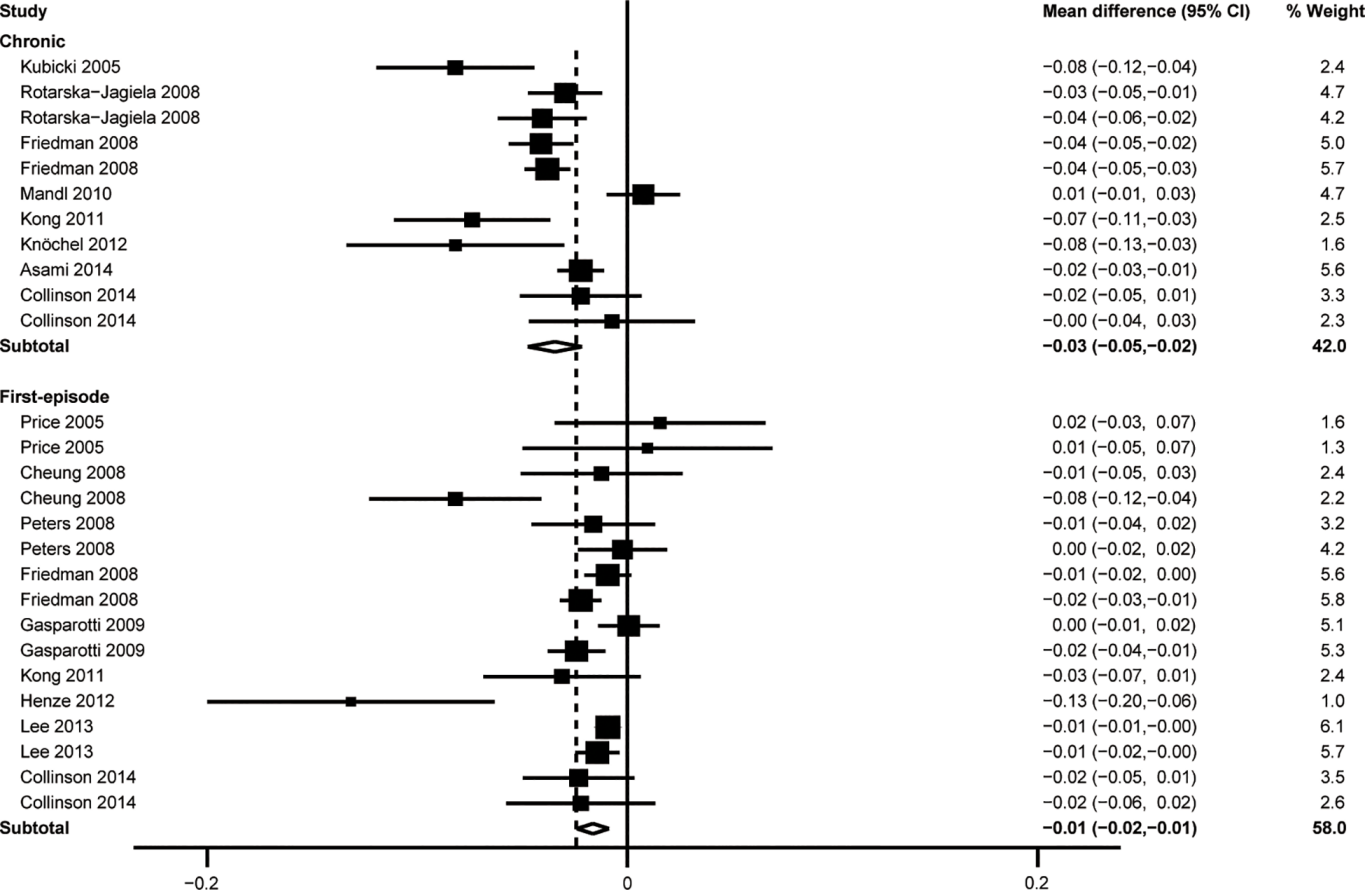
Results of subgroup analysis of different types of schizophrenia.

Subgroup analyses of only first-episode schizophrenic patients (16 comparisons;WMD = -0.014, 95%CI = -0.022–-0.006, Z = 3.00, *P* = 0.001, *I*^2^ = 60.3%) and only chronic schizophrenic patients (11 comparisons; WMD = -0.032, 95%CI = -0.045–-0.019, Z = 4.91, *P* < 0.001, *I*^2^ = 76.7%) revealed that both subgroups had lower FA values in the CC than unrelated healthy controls (**Fig 5**).

## Discussion

The present study demonstrated reduced FA values in the CC of both first-episode and chronic schizophrenia patients, relative to healthy controls.

Our findings are consistent with previous studies showing FA abnormalities in the entire CC of schizophrenic patients[19, 35]. However, the results of studies considering different subregions of the CC as regions of interest have been inconsistent[36]. Nevertheless, findings of WM changes in schizophrenic patients are consistent with other prior findings of schizophrenia-associated loss of WM integrity in principle. Notably, Kunimatsu and colleagues have described reduced expression of genes related to myelin/oligodendrocyte function, oligodendrocyte hypodensity and lamellar abnormalities of myelin as well as abnormal distribution patterns of interstitial neurons in schizophrenic brains[25].

Although schizophrenia presents in late adolescence to early adulthood, it has been theorized to have an extended neurodevelopmental etiology, with brain pathophysiological processes beginning long before the overt manifestation of clinical symptoms[37].Schizophrenia-associated differences in FA values may have embryonic, structural and functional causes. The genu region is formed by axons projecting from the cingulate cortex under the guidance of glia cells, whereas the splenium region develops under the guidance of axons passing from the hippocampal commissure without glial guidance[38].

This meta-analysis study had several limitations that should be acknowledged. Firstly, the study samples were not homogenous. The considerable heterogeneity across the compared datasets may be due to differential patient selection procedures across the included studies. The analyses were further complicated by the fact that imaging methods varied across the analyzed studies. A standardization of the acquisition protocol for DTI in schizophrenia studies would be helpful for alleviating this complication. Secondly, although DTI-derived radial and axial diffusivity are also informative regarding WM integrity, few articles report these data. Consequently, to enable statistical analysis, we focused only on FA data for the CC.

In conclusion, the present DTI findings provide a clear demonstration of a significant overall FA decrease in the WM of the CC in schizophrenic patients. Anatomically, the reduction appeared to be significant in both the genu region and the splenium region. Furthermore, the FA difference of the CC as a whole was pronounced in both chronic schizophrenia patients and those experiencing their first bout with schizophrenia. Further studies with more homogenous populations and standardized DTI protocols are needed to confirm and extend these findings.

## Supporting Information

S1 File PRISMA 2009 Flow Diagram. (DOC)

S2 File Detailed search strategy. The file only contains the search strategy and results of PubMed and Embase.
(DOCX)

S3 File Detailed FA data from included studies. (XLSX)

S1 Checklist PRISMA 2009 Checklist. (DOC)
