# Supplementary material for: Diffusion Tensor MR Imaging Evaluation of Callosal Abnormalities in Schizophrenia: A Meta-Analysis
Source: PLoS One. 2016 Aug 18;11(8):e0161406. doi: 10.1371/journal.pone.0161406 (PMC4990171; doi:10.1371/journal.pone.0161406)
Supplement: S1 File — (DOC) [file pone.0161406.s001.doc]

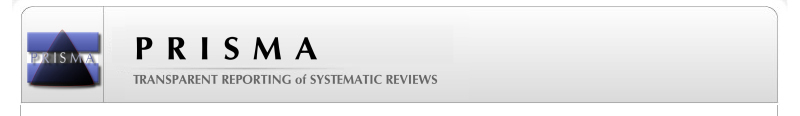
**PRISMA 2009 Flow Diagram**

**Screening**

**Included**

**Eligibility**

**Identification**

Records identified through database searching
(n = 567)

Additional records identified through other sources
(n = 18)

Records after duplicates removed
(n = 678)

Records screened
(n = 215)

Records excluded
(n = 463)

Full-text articles assessed for eligibility
(n = 68)

Full-text articles excluded (n = 147) for reasons:

- Not compare patients with healthy controls (23);

- Not include CC or part of CC as research object (56);

- Not provide FA values (20);

- Other reasons (48);

Studies included in qualitative synthesis
(n = 36)

Studies included in quantitative synthesis (meta-analysis)
(n = 22)
