# Supplementary material for: Diffusion Tensor MR Imaging Evaluation of Callosal Abnormalities in Schizophrenia: A Meta-Analysis
Source: PLoS One. 2016 Aug 18;11(8):e0161406. doi: 10.1371/journal.pone.0161406 (PMC4990171; doi:10.1371/journal.pone.0161406)
Supplement: S2 File — The file only contains the search strategy and results of PubMed and Embase. (DOCX) [file pone.0161406.s002.docx]

# Search strategy and results

## PubMed

#### Search strategy:

(("Schizophrenia"[Mesh] OR Schizophrenia[tiab] OR Schizophrenias[tiab] OR schizophrenic[tiab] OR schizophreniac[tiab] OR Dementia Praecox[tiab] OR "Schizophrenia, Paranoid"[Mesh] OR Delusional Disorder[tiab] OR Delusional Disorders[tiab]) AND ("Corpus Callosum"[Mesh] OR Callosum[tiab] OR Callosums[tiab] OR Neocortical Commissure[tiab] OR Neocortical Commissures[tiab] OR Interhemispheric Commissure[tiab] OR Interhemispheric Commissures[tiab] OR "White Matter"[Mesh] OR White Matter[tiab] OR White Matters[tiab] OR "Brain"[Mesh] OR Brain[tiab] OR Brains[tiab] OR Encephalon[tiab] OR Encephalons[tiab])) AND ("Diffusion Tensor Imaging"[Mesh] OR Diffusion Tensor Imaging[tiab] OR Diffusion Tractography[tiab] OR DTI[tiab])

#### Results: 564

## Embase

#### Search strategy:

(('schizophrenia'/exp OR 'Schizophrenia':ab,ti OR 'Schizophrenias':ab,ti OR 'schizophrenic':ab,ti OR 'schizophreniac':ab,ti OR 'Dementia Praecox':ab,ti OR 'Delusional Disorder':ab,ti OR 'Delusional Disorders':ab,ti OR 'dementia precox':ab,ti) AND ('corpus callosum'/exp OR 'Callosum':ab,ti OR 'Callosums':ab,ti OR 'Neocortical Commissure':ab,ti OR 'Neocortical Commissures':ab,ti OR 'Interhemispheric Commissure':ab,ti OR 'Interhemispheric Commissures':ab,ti OR 'callosal body':ab,ti OR 'corpora callosa':ab,ti OR 'truncus corporis callosi':ab,ti OR 'white matter'/exp OR 'White Matter':ab,ti OR 'White Matters':ab,ti OR 'substantia alba':ab,ti OR 'brain'/exp OR 'Brain':ab,ti OR 'Brains':ab,ti OR 'Encephalon':ab,ti OR 'Encephalons':ab,ti OR 'cerebrum':ab,ti)) AND ('diffusion tensor imaging'/exp OR 'Diffusion Tensor':ab,ti OR 'Diffusion Tractography':ab,ti OR 'DTI':ab,ti OR 'DTT':ab,ti)

#### Results: 529
